# CONTEXTUAL DETERMINANTS OF AGING AND HEALTH

**DOI:** 10.1093/geroni/igae098.0594

**Published:** 2024-12-31

**Authors:** Jiao Yu, Roland Thorpe

**Affiliations:** Chair; Discussant

## Abstract

Addressing social determinants of health is crucial for promoting healthy aging. As this field grows, there is an increasing emphasis on understanding the upstream contextual factors of poor health and inequities. Contextual factors, operating at multiple societal levels, profoundly shape health and well-being among older adults. Guided by social disadvantage theory, this symposium aims to advance research on contextual determinants of aging and health through both innovative multilevel and life course perspectives as well as methodological advancements in novel statistical models and machine learning methods. This symposium will present four studies exploring how important contextual factors, ranging from education, neighborhoods, and childhood social-historical contexts are associated with health outcomes among older adults. Dr. Kim et al.’s interdisciplinary study delves into the micro-level, employing quantile regressions to investigate gender differences in the effects of education on brain pathology and cognitive decline. Dr. Yang et al. explore how meso-level neighborhood affluence and disadvantage influence older adults’ pain outcomes and medication choices. Focusing on minority aging, Dr. Qin et al. examine how neighborhood disadvantage moderates the association between disability and cognitive decline among older Mexican Americans. Finally, Huo and co-authors take a life course perspective, utilizing machine learning tree and forest models to examine a comprehensive set of 43 childhood micro- and meso-level family and residential characteristics contributing to later life frailty. Through these diverse investigations, this session will provide insights into the contextual determinants related to healthy aging, which will inform targeted interventions and policies to address health disparities among older populations.

